# Socioeconomic deprivation and perinatal anxiety: an observational cohort study

**DOI:** 10.1186/s12889-024-20608-4

**Published:** 2024-11-15

**Authors:** Catherine Best, Susan Ayers, Andrea Sinesi, Rose Meades, Helen Cheyne, Margaret Maxwell, Stacey McNicol, Louise R Williams, Fiona Alderdice, Julie Jomeen, Judy Shakespeare, MAP Study Team, Georgina Constantinou, Simon Gilbody, Agnes Hann, Jennifer Holly, Grace Howard, Una Hutton, Rachael Leonard, Debra Salmon, Nazihah Uddin, James Walker, Anna White, Cassandra Yuill

**Affiliations:** 1 Centre for Healthcare and Community Research, Faculty of Health Sciences and Sport, University of Stirling, Stirling, FK9 4LA UK; 2 Centre for Maternal and Child Health Research, School of Health and Psychological Sciences, City, University of London, Northampton Square, London, EC1V 0HB UK; 3 Retired General Practitioner, Oxford, OX2 7AG UK; 4 National Perinatal Epidemiology Unit, Oxford Population Health, Old Road Campus. Headington, Oxford, OX3 7LF UK; 5 Southern Cross University, Gold Coast Airport, Terminal Drive, Bilinga, QLD 4225 Australia

**Keywords:** Perinatal, Anxiety, Pregnancy, Socio-economic factors, Mental health, Ethnicity

## Abstract

**Background:**

Women from areas of social deprivation and minority ethnic groups are more likely to experience poor physical health and have higher rates of mental health problems relative to women from less socially disadvantaged groups. However, very little research has examined this in relation to perinatal anxiety. The current study aims to determine prevalence, risk factors and desire for treatment for perinatal anxiety in three regions of the UK with diverse regional characteristics.

**Methods:**

Women completed measures of anxiety in early, mid-, late-pregnancy and postpartum. Participants were included from three regions of the UK: Region 1 = North East England & North Cumbria *n* = 512; Region 2 = London North Thames *n* = 665; Region 3 = West Midlands *n* = 705.

**Results:**

Prevalence of perinatal anxiety was lower in Region 1 (OR 0.63 95% CI 0.45 to 0.89) and Region 2 (OR 0.72 95% CI 0.52 to 0.98) relative to Region 3. Analysis showed the effect of neighbourhood socioeconomic deprivation on perinatal anxiety differed by region. In more affluent regions, living in a deprived neighbourhood had a greater impact on perinatal anxiety than living in a deprived neighbourhood in a deprived region. Other factors associated with risk of anxiety in the perinatal period included physical health problems and identifying as being from ‘mixed or multiple’ ethnic groups.

**Conclusions:**

Neighbourhood deprivation relative to regional deprivation is a better predictor of perinatal anxiety than either regional deprivation or neighbourhood deprivation alone. Women of mixed ethnic backgrounds and women with physical health problems may warrant more attention in terms of screening and support for perinatal anxiety. Self-reported desire for treatment was found to be low.

**Supplementary Information:**

The online version contains supplementary material available at 10.1186/s12889-024-20608-4.

## Background

The perinatal period from conception to 12 months after birth is a unique and challenging time which impacts on women, infants and their partners and family. It is estimated that perinatal mental health problems affect one in five women with a high associated cost to society. For example, perinatal mental health (PMH) problems cost the United Kingdom (UK) £8.1 billion and the United States of America (USA) $14 billion for every annual cohort of women, with a substantial proportion of this cost due to the long-term impact on the child [4, 15]. Anxiety is a common perinatal mental health condition with an estimated 20.7% of women experiencing an anxiety disorder during the perinatal period [9]. A recent meta-analysis has found similar levels of perinatal anxiety in low- and middle-income countries (22.2%) [22]. Perinatal anxiety is associated with multiple adverse perinatal outcomes for the infant [10]. Relative to perinatal depression, anxiety has received little research attention despite having high prevalence and therefore is the primary focus of this study. Previous studies have identified socioeconomic disadvantage, low social support, a history of mental health problems and previous perinatal complications as risk factors for perinatal anxiety [14] but there have not been any analyses of how regional differences relate to these individual factors.

In many countries most mental health research is conducted in a small number of geographical locations which usually cluster around research institutions, with less activity in more deprived regions where mental illness may be more prevalent. National studies of adult psychiatric morbidity, such as the UK Adult Psychiatric Morbidity Survey (APMS), have not been able to collect enough data on perinatal anxiety in those from ethnic minority and deprived groups to enable analysis [20]. To address this disparity, it is important for research to be conducted in diverse geographic locations to enable comparison between regions with different characteristics (National Institute for Health Research (NIHR), 2021). It is important to provide specific information on perinatal anxiety in these regions to plan PMH services in regions that have higher unmet needs. This could potentially have a positive impact on women and children’s health outcomes, and reduce the significant health, social and economic costs associated with perinatal anxiety.

This paper reports secondary analyses of the prevalence and risk factors for perinatal anxiety from different regions of England, using a UK population-based cohort of 2,243 women who were followed through pregnancy and after birth for the Methods of Assessing Perinatal Anxiety study [1]. The MAP study assessed anxiety symptoms using the Stirling Antenatal Anxiety Scale [2] in all participants and assessed anxiety disorders in a subsample. The MAP cohort provides an opportunity to examine regional differences in the prevalence and correlates of perinatal anxiety. Analyses reported in this paper focus on MAP participants from three regions of the UK: the North East England and North Cumbria (Region 1: *n* = 510) London North Thames (Region 2: *n* = 661) and the West Midlands (Region 3: *n* = 700).

### Aims

The secondary analyses reported here aimed to:

1. Provide information on the prevalence of anxiety at four timepoints during pregnancy and after birth in these regions.
2. Determine the main risk factors for perinatal anxiety in these regions.
3. Provide information on other health and well-being outcomes (depression, psychological distress, quality of life and disability) in these regions.
4. Provide information on support and desire for treatment in these regions.

## Methods

### Study design

The MAP study is an observational cohort design and was conducted between November 2020 and November 2021. Participants were recruited in early pregnancy and were eligible for the MAP cohort if they were: aged 16 years or over; less than 15 weeks pregnant at the time of recruitment; able to provide written informed consent; and had sufficient English to understand and complete questionnaires. Participants completed questionnaire measures of anxiety, depression and psychological distress during early pregnancy (median 12 weeks IQR 11–13), mid-pregnancy (median 23 weeks, IQR 22–24), late pregnancy (median 32 weeks, IQR 31–33) and postpartum (median 7 weeks, IQR 6–9). Participants also provided information on socio-demographic characteristics, previous mental health conditions and physical health conditions at the first timepoint. At all subsequent timepoints they were asked about whether they had received mental health treatment, desire for treatment, quality of life and social support. A subsample of the MAP cohort (*n* = 403) also completed a diagnostic interview to confirm the presence of anxiety disorders and major depressive episodes. The study protocol is available online [18] and the project was pre-registered [1].

### Measures

A number of measures were used in the MAP study to assess sociodemographic characteristics and participants’ symptoms of anxiety and depression, psychological distress, perceived need for treatment, quality of life, physical health, social support and disability. The measures of anxiety and depression, psychological distress, and quality of life were validated and have been previously used with perinatal women.

#### Sociodemographic information

 Was gathered by self-report. Demographic data included age, ethnicity, highest level of education, and marital status. The Index of Multiple Deprivation (IMD) was used to investigate the region-level deprivation of the sample based on participants’ postcodes [17]. The UK Indices of Deprivation measure relative levels of deprivation in over 30,000 small areas or neighbourhoods, and use information on income, employment, education, health deprivation, crime, barriers to housing and services, and living environment to determine relative deprivation.

#### Anxiety

The primary outcome of this study was measured using the Stirling Antenatal Anxiety Scale [24]. This 10-item scale includes both general and pregnancy-specific anxiety symptoms and was found to have good diagnostic accuracy at a cut-off score of 9 or above when compared to a gold standard diagnostic interview [2].

The Whooley questions [28], recommended by the National Institute for Health and Care Excellence (NICE) in England and Wales [19], are used to screen for possible depression. Answering ‘yes’ to one or both questions indicates possible depression.

General psychological distress was assessed using the Clinical Outcomes in Routine Evaluation (CORE-10), a 10-item measure of psychological distress often used in counselling and clinical psychology services in the UK [3].

#### Anxiety disorders

 Were assessed using a gold standard interview for psychiatric disorders: the Mini International Neuropsychiatric Interview version 7.0.2 (MINI) [23]. Disorders were recorded if they were present at the time of the interview. Inter-rater reliability was checked for 5% of interviews selected at random and was 96%.

#### Treatment for psychological problems

 Was measured by asking participants to answer yes, no, or not applicable to the following: whether they had received professional help or treatment for current mental health or psychological problems and ‘If you are currently experiencing psychological problems, is this something you would like professional help or treatment for?’.

#### Health-related quality of life

 Was measured with the EQ-5D-5L [12] at each time point. The EQ-5D-5L includes a measure of general health status using a visual analogue scale ranging from 0 to 100. Disability associated with health conditions was assessed using a single question ‘If you have health problems, how much do they interfere with your day-to-day activities (e.g. work, study, housework, family or leisure activities)? To which the response options were ‘most of the time’, ‘some of the time’, ‘rarely’ or ‘never’. Disability associated with mental health conditions was assessed with the question ‘If, in these [anxiety questionnaires] you indicated that you have problems, how difficult have these problems made it for you to do your work, take care of things at home, or get along with other people?’ to which the response options were ‘not at all difficult’, ‘somewhat difficult’, ‘very difficult’ or ‘extremely difficult’.

#### Physical health

 Was assessed at the first timepoint only, through the single question “Do you have existing health conditions?” with responses of yes, no, and don’t know, and the possibility to indicate a specific health condition from a number of options.

#### Social support

 Was measured as a continuous variable through the ENRICHD social support instrument (ESSI) which is a 7 item self-report instrument that assesses four elements of social support: emotional, instrumental, informational and appraisal [7].

### Analysis

Sociodemographic characteristics are presented by region. Differences between regions were tested using the Chi Square test for categorical variables and regression for continuous variables.

Descriptive statistics for the primary outcome, prevalence of anxiety, and for secondary outcomes of depression, psychological distress, quality of life and disability are presented by region and stage of the perinatal period.

The proportion of women who meet the threshold for perinatal anxiety was defined by a cut off of 9 or more on the SAAS [2]. Associated 95% confidence intervals for this proportion are reported or indicated by error bars.

For continuous outcomes, such as EQ-5D-5L visual analogue scale, the mean and standard deviation by region and perinatal stage are reported.

Differences by region were assessed using generalised linear mixed models appropriate for the distribution of the outcome i.e. logit for binary outcomes. The independent variable was region with Region 3 as the reference category. These models were adjusted for perinatal stage. More detail on the analysis models is presented in the Supplementary materials.

The relationship between sociodemographic and health factors and the outcome of perinatal anxiety was explored using generalised linear mixed models. Perinatal stage was included as a covariate with a random intercept at the individual participant level was included in the model. Analysis was conducted in Stata version 17.

## Results

### Sample characteristics

The characteristics of participants within each of the three regions is shown in Table 1. The mean age of participants was 31.3 years (SD 5.2, range 16–50). Mean age was lower in Region 1 (29.4 years, sd 5.4) than in either region 2 (32.1 years, sd 4.9) or Region 3 (31.8 years, sd 5.0).

**Table 1:** Sample characteristics by region^a^

			Location				
		N	Region 1^b^n (%)	Region 2^b^n (%)	Region 3^b^n (%)	Total n (%)	Chi Square
Ethnic group	White ethnic group	1674	436 (94.99%)	343 (57.55%)	454 (73.34%)	1233 (73.66%)	187.35 *p* < 0.001
	Other ethnic group		23 (5.01%)	253 (42.45%)	165 (26.66%)	441 (26.34%)	
Highest level of education	Less than degree level	1677	258 (55.97%)	168 (28.09%)	226 (36.57%)	652 (38.88%)	87.30 *p* < 0.001
	Degree level or more		203 (44.03%)	430 (71.91%)	392 (63.43%)	1025 (61.12%)	
Ever experienced psychological/mental health problems	Don’t know	1795	15 (3.11%)	40 (6.18%)	34 (5.11%)	89 (4.96%)	28.35 *p* < 0.001
	No		276 (57.26%)	439 (67.85%)	397 (59.61%)	1112 (61.95%)	
	Yes		191 (39.63%)	168 (25.97%)	235 (35.29%)	594 (33.09%)	
Received professional help or treatment for these psychological or mental health problems	No	538	18 (11.32%)	17 (10.43%)	26 (12.04%)	61 (11.34%)	1.32 *p* = 0.858
	Yes, currently		37 (23.27%)	31 (19.02%)	43 (19.91%)	111 (20.63%)	
	Yes, in the past		104 (65.41%)	115 (70.55%)	147 (68.06%)	366 (68.03%)	
Pre-existing health condition	No	1765	360 (74.69%)	515 (83.06%)	465 (70.14%)	1340 (75.92%)	29.85 *p* < 0.001
	Yes		122 (25.31%)	105 (16.94%)	198 (29.86%)	425 (24.08%)	

^a^Sample characteristics were measured in early pregnancy

^b^Region 1 = North East England & North Cumbria; Region 2 = London North Thames; Region 3 = West Midlands

The proportion of participants from a white ethnic background differed between regions. Region 2 was more ethnically diverse than the other two regions. Regions also differed in terms of proportion of women with pre-existing health conditions. Region 2 had lower levels of pre-existing health conditions and lower levels of previous experience of mental health problems compared to the other two regions.

**Fig. 1:**
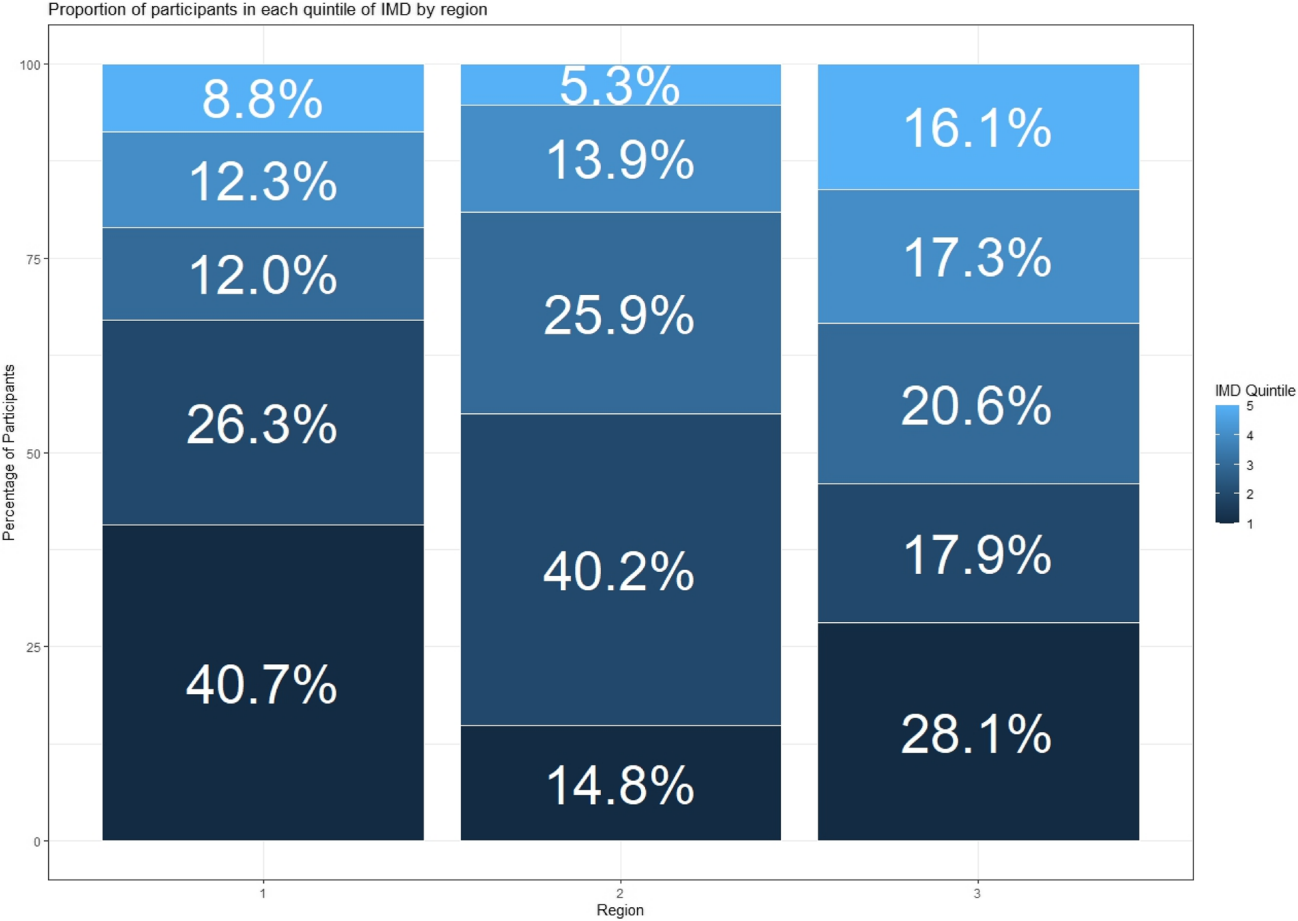
Proportion of participants in each quintile of IMD by region

Figure 1 shows the proportion of participants from each region in each quintile of IMD at the early pregnancy time point. In early pregnancy, 46.0% of Region 3 participants, 67.0% of Region 1 participants and 55.0% of Region 2 participants were in the two most deprived quintiles of IMD. This difference between regions is statstistically significant (χ^2^ (2) = 46.82 *p* < 0.001).

### Prevalence of anxiety

**Fig. 2:**
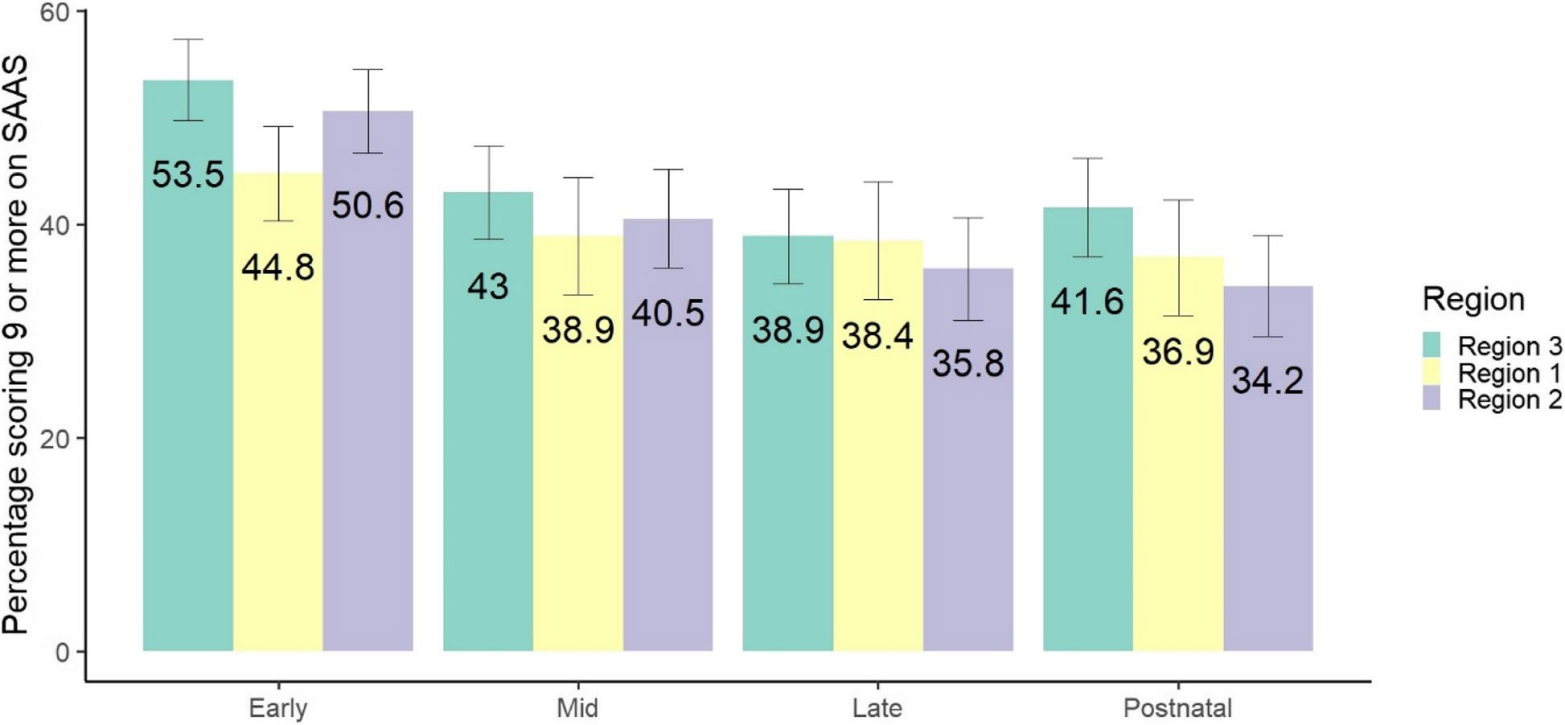
Prevalence of self-reported anxiety by region and timepoint (*n* = 1849)

The prevalence of anxiety using a cut-off of 9 on the SAAS questionnaire is shown in Fig. 2 for each timepoint across regions. The distribution of total scores on the SAAS at the early pregnancy timepoint is given in supplementary files (see Fig. 1e). The marginal predicted probabilities of scoring over 9 on the SAAS is 46.0% (95% CI 42.9 to 49.0) in Region 3, 39.7% (95% CI 36.3–43.2%) in Region 1 and 41.3% (95% CI 38.3 to 44.5) in Region 2. A mixed effects logistic regression analysis exploring perinatal anxiety (as defined by a score of 9 or more on the SAAS) by region, adjusted for perinatal stage, indicated participants were less likely to have anxiety in Region 1 (OR 0.63; 95% CI 0.45 to 0.89) and Region 2 (OR 0.72; 95% CI 0.52 to 0.98) relative to Region 3.

The same pattern of prevalence was found in the subsample of participants who completed the MINI diagnostic interview (Table 2), with the highest prevalence for any anxiety diagnosis in Region 3.

**Table 2:** Prevalence of anxiety disorders at each timepoint in different regions

	Early pregnancy	Mid pregnancy	Late pregnancy	Post-natal	Total
	*N* = 71	*N* = 89	*N* = 99	*N* = 89	*N* = 348
Region 1^a^	15.4%	20.0%	8.3%	18.8%	16.4%
Region 2^a^	20.8%	25.0%	6.5%	0.0%	13.2%
Region 3^a^	29.4%	18.6%	19.6%	29.3%	23.2%

^a^Region 1 = North East England & North Cumbria; Region 2 = London North Thames; Region 3 = West Midlands

### Prevalence of depression and psychological distress

Table 3 reports the percentage of participants who met criteria on a screening tool for depression and the percentage of participants who met criteria on a screening tool for general psychological distress at each perinatal stage by region. The table also shows the mean scores for health-related quality of life and social support at each perinatal stage by region. Table 4 shows disability due to psychological and physical problems as reported by participants in different regions. Rates of depression, psychological distress were all reduced in Regions 1 and 2 relative to Region 3, although these differences were only statistically significant for the lower rate of depression in Region 1 compared to Region 3. Health related Quality of Life and social support were higher in Regions 1 & 2 relative to Region 3. Again, these differences were only statistically significant in Region 1 compared to Region 3. There were no statistically significant differences between Region 2 and the Region 3 on any of these measures across all perinatal stages combined. Particpants in Region 1 had lower levels of interference with activities of day to day life both from problems resulting from health conditions and problems resulting from mental health conditions. There were no differences between Region 2 and Region 3.

**Table 3:** Depression, distress, quality of life and social support in different regions

		Early pregnancy	Mid pregnancy	Late pregnancy	Postnatal	Total	Odds ratio^a^
		%	%	%	%	%	
Depression	Region 3	47.06	34.92	30.55	34.33	37.85	ref
	Region 1	37.35	31.05	29.97	29.14	32.62*	0.65 (0.49 to 0.86) *p* = 0.002
	Region 2	47.93	32.87	28.57	28.39	36.36	0.90 (0.70 to 1.16) *p* = 0.409
	N	1940	1301	1223	1190	5657	
Psychological distress	Region 3	33.78	29.13	27.59	25.46	29.54	ref
	Region 1	29.34	27.39	26.69	22.19	26.79	0.76 (0.55 to 1.06) *p* = 0.110
	Region 2	34.70	27.93	25.33	21.20	28.33	0.98 (0.72 to 1.34) *p* = 0.918
	N	1900	1294	1219	1189	5602	
		Mean score	Mean score	Mean score	Mean score	Mean score	Mean difference^§^
Health-related quality of life (VAS score)	Region 3	80.13	78.77	76.87	77.60	78.59	ref
	Region 1	81.39	80.19	78.52	81.31	80.51*	2.04 (0.43 to 3.64) *p* = 0.013
	Region 2	79.16	79.87	80.56	79.18	79.61	0.96 (-0.52 to 2.45) *p* = 0.204
	N	1763	1140	1026	986	4915	
Social support	Region 3	31.31	31.06	31.17	30.79	31.12	ref
	Region 1	32.06	31.21	31.45	31.44	31.62*	0.58 (0.15 to 1.00) *p* = 0.008
	Region 2	31.29	31.25	31.58	31.06	31.30	0.04 (-0.36 to 0.44) *p* = 0.849
	N	1645	1067	973	919	4604	

Depression = Whooley questions; Psychological distress = CORE-10, QoL = EQ 5D 5 L VAS, Social support = ESSI. Region 1 = North East England & North Cumbria, Region 2 = London North Thames, Region 3 = West Midlands

^a^From GLMM adjusted for perinatal stage and with random intercept for individual see supplementary file for more detail

*p < 0.05 for scores across all timepoints relative to Region 3

**Table 4:** Disability associated with health conditions and psychological problems

		Early pregnancy	Mid pregnancy	Late pregnancy	Post-natal	Total	Odds ratio§ (95% CI) *p* value
Health interferes everyday life (%)	Region 3	43.90	47.12	45.05	36.16	43.27	ref
	Region 1	38.01	41.88	41.41	27.06	37.21*	0.69 (0.53 to 0.90) *p* = 0.006
	Region 2	39.82	41.53	43.64	31.61	39.30	0.82 (0.65 to 1.04) *p* = 0.109
	N	1838	1225	1137	1126	5326	
Psychological problems never interfere (%)	Region 3	58.42	63.31	59.82	69.25	62.19	ref
	Region 1	65.39	69.59	66.43	68.00	67.11*	0.63 (0.46 to 0.87) *p* = 0.005
	Region 2	56.06	66.27	64.27	68.85	62.78	1.00 (0.74 to 1.33) *p* = 0.964
	N	1774	1104	1191	1108	5177	

### Factors associated with perinatal anxiety

Generalised linear mixed models were used to examine which factors were associated perinatal anxiety and to investigate why Region 3 had higher a prevalence of perinatal anxiety than the other two regions. The impact of small area-level deprivation was explored through the IMD of each participant’s postcode of residence.

Inclusion of IMD as a covariate (Table 5, model 2) did not eliminate the difference between regions in terms of the odds of reaching the threshold for anxiety on the questionnaire measure. However, inclusion of an interaction effect between region and IMD lead to the a reduction in the effect size and the difference between regions was no longer statistically significant (Table 5). Model 3 in Table 5 indicates that high deprivation (IMD 1 or 2) increases the odds of reaching the threshold for anxiety in Region 3 but not in the other two regions.

**Table 5:** Effect of socioeconomic deprivation (IMD) in each region on perinatal anxiety

	Model 1 OR [95% CI]	Model 2 OR [95% CI]	Model 3 OR [95% CI]
Region 3	ref	ref	ref
Region 1	0.678^*^	0.656^*^	0.858
	[0.476,0.966]	[0.458,0.939]	[0.495,1.487]
Region 2	0.664^*^	0.654^*^	0.891
	[0.472,0.935]	[0.464,0.921]	[0.551,1.442]
Early pregnancy	ref	ref	ref
Mid pregnancy	0.517^***^	0.518^***^	0.519^***^
	[0.415,0.644]	[0.416,0.646]	[0.416,0.647]
Late pregnancy	0.415^***^	0.417^***^	0.418^***^
	[0.331,0.522]	[0.331,0.524]	[0.333,0.525]
Postnatal	0.396^***^	0.397^***^	0.398^***^
	[0.314,0.499]	[0.316,0.501]	[0.316,0.502]
Low deprivation		ref	ref
High deprivation		1.170	1.659^*^
		[0.874,1.566]	[1.049,2.624]
Region 3 # Low deprivation			ref
Region 3 # High deprivation			ref
Region 1 # Low deprivation			ref
Region 1 # High deprivation			0.592
			[0.286,1.227]
Region 2 # Low deprivation			ref
Region 2 # High deprivation			0.531
			[0.268,1.050]
Variance of the random effect	193.144^***^	189.853^***^	185.115^***^
Observations	4661	4661	4661
*BIC*	5551.731	5559.100	5572.290

We also investigated whether any of the other sociodemographic variables were influential in predicting the prevalence of perinatal anxiety. Table 6, Models 1 and 2, indicate that women from mixed or multiple ethnic backgrounds were significantly more likely to meet the threshold for anxiety relative to women from white ethnic backgrounds, in a model adjusted for perinatal stage and region. Models 1 and 2 indicate that either the inclusion of IMD and an interaction between IMD and region, or the inclusion of IMD and ethnic group are sufficient to remove any differences between the regions in the odds of perinatal anxiety. This may suggest that differences between regions in sociodemographic composition are responsible for the differences between regions in probability of having perinatal anxiety.

In Table 6, Model 3 we included ‘presence of an existing health condition’ and ‘level of social support’ as predictors of perinatal anxiety. Both these factors were found to have a very strong association with perinatal anxiety. In early pregnancy 45.4% of women without an existing health condition met criteria compared to 59.3% of those with an existing health condition.

**Table 6:** Influence of sociodemographic factors on perinatal anxiety

	Model 1 OR [95% CI]	Model 2 OR [95% CI]	Model 3 OR [95% CI]
Early pregnancy	ref	ref	ref
Mid pregnancy	0.523^***^	0.473^***^	0.479^***^
	[0.417,0.656]	[0.370,0.605]	[0.373,0.615]
Late pregnancy	0.421^***^	0.383^***^	0.383^***^
	[0.332,0.533]	[0.296,0.494]	[0.294,0.499]
Postnatal	0.395^***^	0.355^***^	0.352^***^
	[0.311,0.502]	[0.273,0.463]	[0.269,0.461]
Region 3	ref	ref	ref
Region 1	0.874	0.874	0.898
	[0.495,1.544]	[0.593,1.289]	[0.625,1.291]
Region 2	0.923	0.904	0.856
	[0.557,1.528]	[0.625,1.308]	[0.609,1.202]
Low deprivation	ref	ref	ref
High deprivation	1.704^*^	1.108	0.920
	[1.036,2.803]	[0.808,1.520]	[0.685,1.235]
Region 1 # High deprivation	0.613		
	[0.285,1.318]		
Region 2 # High deprivation	0.514		
	[0.251,1.051]		
White	ref	ref	ref
Mixed/multiple ethnic groups	2.658^**^	2.117^*^	3.332^***^
	[1.305,5.412]	[1.016,4.410]	[1.676,6.625]
Asian/Asian British	1.018	0.953	1.361
	[0.630,1.646]	[0.588,1.546]	[0.860,2.153]
Black/African/Caribbean/Black British	0.852	0.414^*^	0.873
	[0.418,1.735]	[0.195,0.878]	[0.433,1.759]
Other ethnic group	0.343	0.298	0.420
	[0.087,1.346]	[0.072,1.229]	[0.106,1.659]
Education-degree	1.050	1.395^*^	1.000
	[0.759,1.452]	[1.003,1.940]	[1.000,1.000]
Social support		0.769^***^	1.189
		[0.740,0.799]	[0.875,1.615]
Any health condition		3.164^***^	
		[2.241,4.466]	
EQ-5D-5 L VAS			0.960^***^
			[0.953,0.967]
Ever experienced psychological/mental health problems			6.092^***^
			[4.432,8.375]
Previous pregnancy loss			1.330
			[0.980,1.806]
Variance of random effect	176.997^***^	97.375^***^	26.562^***^
Observations	4342	3965	3800
*BIC*	5213.114	4522.798	4086.821

Exponentiated coefficients; 95% confidence intervals in brackets

^*^p < 0.05, ^**^p < 0.001, ^***^p < 0.001

Finally, we looked at the influence of previous mental health conditions on the likelihood of perinatal anxiety. Previous mental health conditions had a statistically significant association with perinatal anxiety in models with and without other sociodemographic variables. We examined interaction effects between all the covariates (ethnic group, history of psychological problems, social support, educational level, health status, existing health conditions and previous pregnancy loss) and region to determine whether the effects of these predictors varied by region. There were no significant effects except for the relationship with IMD reported above.

### Desire for professional help or treatment for psychological problems

**Fig. 3:**
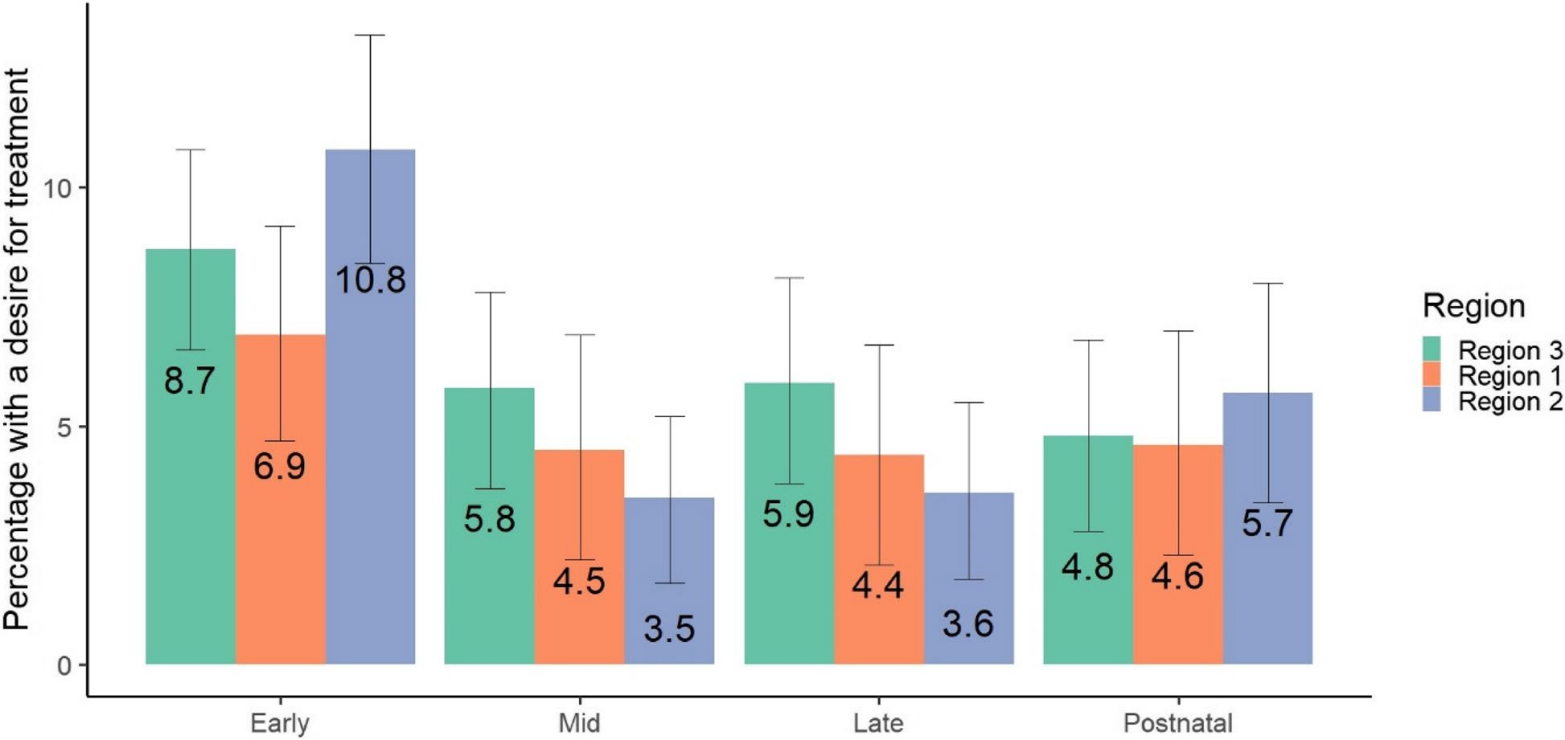
Desire for treatment by region and timepoint (*N* = 1849)

Desire for professional help or treatment was very low in this sample with only 344 (6.05%) of responses indicating desire for treatment for mental health problems across all perinatal stages combined. The proportion of participants who wanted treatment at each perinatal stage in each region is shown in Fig. 3. There were no statistically significant differences between regions in the proportion of participants reporting a desire for treatment.

Region was not associated with desire for treatment in an unadjusted model. In a generalised linear mixed model on desire for treatment adjusted for timepoint, region, ethnic group, education, and category of IMD (low versus high), only IMD was associated with desire for treatment. The adjusted odds for wanting treatment were higher (aOR 2.30; 95% CI 1.14 to 4.62) in women living in neighbourhoods of higher deprivation as measured by the IMD.

## Discussion

The study found that prevalence of anxiety differed by region in a completely unexpected way. The area with the lowest proportion of women living in deprived neighbourhoods had the highest prevalence of anxiety. This contrasts with previous research which finds that low socioeconomic status is associated with greater risk of antenatal anxiety and depression [25]. However, the current study suggests the relationship was complex in that the effect of neighbourhood socioeconomic status depended on context. Our findings suggest that being in a more deprived local neighbourhood within a relatively affluent region has greater negative effects on perinatal mental health outcomes than being in a more deprived local neighbourhood in a more deprived region.

This implies social factors such as social norms, social comparisons and social isolation are likely to be important as has been found in previous work [16] (Guardino and Schetter 2022). If a pregnant woman’s circumstances are like others in their region, then it is their social norm and harder to make negative social comparisons of their circumstances with others around them. They also might be less socially isolated than pregnant women living in relative deprivation in an affluent area. Thus, any impact of social deprivation on perinatal mental health maybe lessened for women living in neighbourhoods in deprived regions compared to those living in deprived circumstances in affluent regions.

The way in which deprivation and disease is measured is also critical. In previous research deprivation was measured using various proxy measures such as level of education or household income, or compound measures such as IMD. Similarly, disease prevalence can be measured in various ways, regions can be defined differently, and therefore results will differ accordingly. For example, Bower et al. [6] used the UK Quality and Outcomes Framework (QOF) disease registers from general practice to assess prevalence of mental illness in the general population and found a similar pattern to regional differences observed in this study in that Region 2 had a lower prevalence of mental health disease than Region 1 or Region 3 [6]. In this study, the modelling uses scores on the Stirling Antenatal Anxiety Scale (cut off 9) as dependent variable and while this score indicates that further assessment maybe required, it is not diagnostic. There were high rates of women meeting this threshold with around 40% of women scoring nine or over on the Stirling Antenatal Anxiety Scale. A strength of this study is that we also present findings from the full diagnostic interview broken down by region, although detailed analysis is not feasible in this smaller diagnostic interview sample.

Findings about other risk factors are consistent with previous research. The finding that previous mental health problems and lack of social support were associated with greater psychological distress during the perinatal period is consistently found in previous research and meta-analyses [5]. Furthermore, the association between ethnic minority status and perinatal anxiety is consistent with previous research [26]. These effects did not differ by region. Similarly, the impact of existing health conditions on the likelihood of experiencing perinatal anxiety is in keeping with a large body of evidence showing an association between physical and mental health [21] in general populations and in relation to satisfaction with maternity care outcomes [8].

Desire for treatment was low in this sample, which is consistent with findings from studies that suggest a proportion of women do not access treatment. For example, Koire and colleagues [13] found that 30% of women who received a prenatal diagnosis of Generalized Anxiety Disorder did not receive any treatment. Similarly, Henshaw and colleagues [11] found although more than 80% of women with perinatal psychopathology discussed concerns about their mental health with their partner only half of them approached a health professional to discuss their difficulties. This indicates help seeking behaviour is low in this group. This may be due to several factors. A recent review of barriers to women accessing perinatal mental healthcare services identified individual factors such as lack of knowledge or negative beliefs about mental illness, healthcare professionals and healthcare services, fear of being judged, logistical barriers, family and social factors (e.g. support or discouragement) and sociodemographic factors [27]. These barriers are unlikely to vary greatly between regions, so it is therefore not surprising that there were no regional differences in desire for treatment. Many women experience anxiety during pregnancy and the early months after birth as this is a time of huge adjustment and change and large number of these women may consider their anxiety normal and assume it is part of the adjustment process and will improve with time. It is also important to note that this study measured *desire for professional help or treatment*, not *need* for treatment or access to treatment. It is therefore possible some of those who would benefit from help did not want to access this through formal services but may seek it elsewhere.

### Strengths and limitations

This study has significant strengths: use of the novel perinatal anxiety-specific Stirling Antenatal Anxiety Scale; measures of important factors, such as social support, physical health, and quality of life and use of a large population-based cohort. This study was large enough that it enabled us to examine regional differences in perinatal anxiety and associated outcomes. However, various limitations also need to be considered. The first is that data were collected during the COVID19 pandemic so regional differences may have been altered or masked through peoples’ responses to the pandemic, particularly in the context of pregnancy because maternity services implemented various restrictions to antenatal and birth care. A second limitation is that the sample were highly educated, with just over 60% being educated to degree level or higher. It is therefore important to examine regional differences in perinatal mental health in more diverse samples. A final limitation is that there is substantial comorbidity between anxiety, depression and other perinatal mental health conditions and it was beyond the scope of this paper to examine how the associations noted between perinatal anxiety and socioeconomic factors compared to those for perinatal depression or other perinatal mental health conditions.

## Conclusions

Results indicate a complex relationship between regional deprivation and risk for perinatal anxiety. Regional differences in the prevalence of perinatal anxiety were largely explained by the sociodemographic composition of regions i.e. in terms of neighbourhood deprivation and ethnic composition. The findings need further exploration and replication in more diverse samples to determine the underlying mechanisms.

## Supplementary Information

Supplementary Material 1.

## Data Availability

Individual participant-level data are not available but authors can provide sample-level data and information on request, after publication. The study protocol is available here https://njl-admin.nihr.ac.uk/document/download/2034506.
