# Supplementary material for: Socioeconomic deprivation and perinatal anxiety: an observational cohort study
Source: BMC Public Health. 2024 Nov 15;24:3183. doi: 10.1186/s12889-024-20608-4 (PMC11566135; doi:10.1186/s12889-024-20608-4)
Supplement: Supplementary file 1 — Supplementary Material 1. [file 12889_2024_20608_MOESM1_ESM.docx]

Supplementary file WP4 paper

Modelling strategy

The models used in the paper are described below.

For comparison of prevalence of anxiety between regions

The main manuscript states ‘Prevalence of perinatal anxiety was lower in Region 1 (OR 0.63 95% CI 0.45 to 0.89) and Region 2 (OR 0.72 95% CI 0.52 to 0.98) relative to Region 3 region.’

This was estimated using generalised linear mixed effects models which can be written as:

g(θ)=Xβ+Zγ

As anxiety is binary (SAAS>8) we use a logistic link function g(.)=log_e_(p/1-p)

The fixed part of the model is Xβ. X is a N x p matrix of the predictor variables- in the case of this comparison these are the independent variable ‘region’ and the covariate ‘perinatal stage’ ; β is a p x 1 column vector of the fixed-effects regression coefficients; Z is the N x q design matrix for the random effects. The model for the comparison of anxiety across regions contains a random intercept for participants parameterised as γ∼Nm(0,σ^2^γI) the rows of Z then indicate which are the same participant.

In Stata

melogit SAAS_cut1 i.area i.time ||ID2:, eform

**Table 3.** Depression, distress, quality of life and social support in different regions

The same model is used as above except that the link function is ‘identity’ as the outcomes are continuous.

**Table 4.** Disability associated with health conditions and psychological problems

Same model as for comparison of prevalence of anxiety between regions

Figure 1e Violin plot of SAAS total score at time point 1

The graph above shows violin plots of the distribution of total anxiety scores in early pregnancy in each region.
